# How to manage ventricular arrhythmia in patients with viral myocarditis

**DOI:** 10.1093/ehjopen/oeae005

**Published:** 2024-02-05

**Authors:** Naoya Kataoka, Teruhiko Imamura

**Affiliations:** Second Department of Internal Medicine, University of Toyama, 2630 Sugitani Toyama, Toyama 930-0194, Japan; Second Department of Internal Medicine, University of Toyama, 2630 Sugitani Toyama, Toyama 930-0194, Japan

**The authors response is available at https://doi.org/10.1093/ehjopen/oeae006.**

Peretto *et al*.^1^ conducted an investigation into the clinical attributes and risk stratification concerning patients experiencing viral myocarditis complicated by either monomorphic ventricular arrhythmia (VA) or polymorphic VA. Their findings revealed that polymorphic VA correlated with ongoing systemic infection and early adverse outcomes. In contrast, monomorphic VA indicated chronic cardiomyopathy and a higher incidence of major adverse events during the mid-term observation period.

They accounted for non-sustained VAs, including ventricular ectopic beats, as VA events.^1^ However, sustained VAs leading to electrical storms bear greater clinical relevance.^2^ Is there a possibility of differentiating between sustained and non-sustained VAs?

The authors established that replacement fibrosis was more prevalent in patients exhibiting monomorphic VA compared to those with polymorphic VA.^1^ It is conceivable that re-entry VA may develop in individuals with advanced myocardial fibrosis during the chronic phase. Nonetheless, the authors solely considered VAs that manifested within 24 h following hospitalization. The progression of myocardial fibrosis typically requires time.^3^ How do the authors reconcile the temporal discrepancy between early VAs and myocardial fibrosis?

One plausible explanation could involve the interval between the onset of myocarditis and the index admission. In the monomorphic VA group, the progression of myocarditis might have been more gradual, and the duration between disease onset and the index admission possibly longer.

In the authors’ study, a lower left ventricular ejection fraction (LVEF) upon admission did not correlate with future clinical outcomes.^1^ However, in patients with typical myocarditis, LVEF usually gradually deteriorates to its lowest levels. Did the authors take into account the prognostic implications of such a trajectory of LVEF?

Finally, how do the authors contemplate the clinical implications of their findings? A critical clinical inquiry revolves around the indication of implantable cardiac defibrillators in patients with myocarditis and VA. Considering their discovery that monomorphic VA was linked with recurrent major VA during mid-term follow-up,^1^ it suggests the potential indication of an implantable cardiac defibrillator for such a cohort.

## Data Availability

No new data were generated or analysed in support of this research.
